# ﻿*Gastrochilusbalangshanensis* (Orchidaceae, Aeridinae), a new subalpine epiphytic orchid from the Mountains of Southwest China

**DOI:** 10.3897/phytokeys.247.130755

**Published:** 2024-10-11

**Authors:** Jun-Yi Zhang, Yue-Hong Cheng, Min Liao, Sen-Long Jin, Hong-Qiang Lin, Pan-Yan Yang, Hai He, Bo Xu

**Affiliations:** 1 CAS Key Laboratory of Mountain Ecological Restoration and Bioresource Utilization & Ecological Restoration and Biodiversity Conservation Key Laboratory of Sichuan Province, Chengdu Institute of Biology, Chinese Academy of Sciences, Chengdu 610041, China Chengdu Institute of Biology, Chinese Academy of Sciences Chengdu China; 2 University of Chinese Academy of Sciences, Beijing 10049, China University of Chinese Academy of Sciences Beijing China; 3 Wolong National Natural Reserve Administration Bureau, Wenchuan 623006, Sichuan, China Wolong National Natural Reserve Administration Bureau Wenchuan China; 4 College of Life Sciences, Chongqing Normal University, Chongqing 401331, China Chongqing Normal University Chongqing China

**Keywords:** Hengduan Mountains, new species, phylogeny, Sichuan, Vandeae

## Abstract

*Gastrochilusbalangshanensis*, a new orchid species from the Balang Mountain, Sichuan Province, Southwest China, is described and illustrated. It morphologically resembles *G.affinis*, but differs in having shorter stems, a reniform epichile and a sub-hemispherical hypochile (spur), obtuse-rounded at the apex. The results of molecular phylogenetic analyses based on nuclear ribosome internal transcribed spacer (nrITS) and four chloroplast DNA markers (*mat*K, *psb*A–*trn*H, *psb*M–*trn*D and *trn*L–F) from 50 *Gastrochilus* species indicate that *G.balangshanensis* is closely related to *G.heminii* and *G.bernhardtianus*, also endemic to the Hengduan Mountains. The novelty is a branch and trunk epiphyte in mixed coniferous forest.

## ﻿Introduction

*Gastrochilus* D.Don (Don 1825) is a vandoid genus of epiphytic orchids, within subtribe Aeridinae, consisting of 79 species, widely distributed in Tropical and Subtropical Asia (Tsi 1996; Tsi 1999; Liu et al. 2019; Govaerts et al. 2021; Zhou et al. 2021; Zhang et al. 2022; Ya et al. 2023; Zhang et al. 2024). It is characterized by the enlarged and saccate hypochile, forming a spur, and two subglobose pollinia borne on a slender stipe (Pridgeon et al. 2014; Liao et al. 2022). Recently, based on molecular and morphological data, a new infrageneric classification of *Gastrochilus* has been proposed by Zhang et al. (2024), dividing the genus into six sections, viz. *G.* sects. *Acinacifolii* Q.Liu & J.Y.Gao ex Jun Y.Zhang & H.He, *Brachycaules* Q.Liu & J.Y.Gao ex Jun Y.Zhang & H. He, *Caespitosi* Z.H.Tsi, *Gastrochilus*, *Microphylli* (Benth. & Hook.f.) Seidenf. and *Pseudodistichi* Jun Y.Zhang & H.He. More than 20 new species of *Gastrochilus* were described in the past five years, greatly enriching the diversity of this genus (Liu et al. 2019; Rao et al. 2019; Wu et al. 2019; Chen et al. 2022; Dey et al. 2022; Liao et al. 2022; Nguyen et al. 2022; Zhang et al. 2022; Lee et al. 2023; Liu et al. 2023; Ya et al. 2023; Zhang et al. 2024; Zhou et al. 2024).

In April 2023, during a survey of orchid diversity in the subalpine forests of the Balang Mountain in Sichuan Province, Southwest China, some flowering specimens of *Gastrochilus* were collected. They were tentatively ascribed to the sectionMicrophylli, which is characterized by the closely alternate leaves and a smooth-glabrous epichile (Zhang et al. 2024). After detailed morphological examination, the material could not be assigned to any recognized species of G.sect.Microphylli. Our phylogenetic analyses, combining the nuclear ribosome internal transcribed spacer (nrITS) with four plastid markers of 49 congeneric taxa, also support its recognition as a new species, which we describe here.

## ﻿Materials and methods

### ﻿Morphological analyses

Herbarium specimens and silica-gel dried leaves of the novelty were collected in the field in the Balang Mountain, Wenchuan County, Sichuan Province, Southwest China. The measurements and description of the novelty were based on four field-collected living plants and three dried herbarium specimens (*Jun-Yi Zhang & Yue-Hong Cheng ZJY185*; *Jun-Yi Zhang & Yue-Hong Cheng ZJY186*; *Jun-Yi Zhang & Yue-Hong Cheng ZJY204*). The taxonomic description follows the terminology used by Beentje (2016). Voucher specimens and additional silica-gel dried leaves are deposited in CDBI Herbarium (acronym following Thiers 2021, continuously updated). Additionally, we examined the scans of six specimens of three closely related taxa, including relevant type specimens (see taxonomic treatment for details), deposited at CDBI, K, PE and KUN.

### ﻿DNA extraction, amplification and sequencing

The sequences of the 56 species included in the molecular phylogenetic analysis, originally published in Liu et al. (2019) and Zhang et al. (2024), were retrieved from GenBank, except those obtained from two individuals of the new species, which were newly generated in this study. Detailed information concerning the DNA markers, sampled taxa, voucher collections and GenBank accession numbers are listed in Appendix 1: Tables A1, A2. Total DNA was extracted exclusively from silica-gel dried leaves via a Plant DNA Isolation Kit (Cat.No.DE-06111). Based on the phylogenetic studies of *Gastrochilus* by Liu et al. (2019) and Zhang et al. (2024), we applied the same primers to amplify its nuclear ribosome internal transcribed spacer (nrITS) and four chloroplast DNA fragments (*mat*K, *psb*A–*trn*H, *psb*M–*trn*D, and *trn*L–F) through polymerase chain reaction (PCR). All DNA samples were sent to TSINGKE Biotech Co. Ltd (Chengdu, China) for sequencing. The final manually corrected sequences were then submitted to GenBank (Appendix 1: Table A2).

### ﻿Phylogenetic analyses

All sequences were edited via Sequencher v4.1.4 (Gene Codes, Ann Arbor, Michigan, USA) and aligned via MAFFT v7.475 (Katoh and Standley 2013) with default parameters. We performed phylogenetic analyses based on the datasets of combined nuclear ribosome internal transcribed spacer (nrITS) and the four chloroplast DNA fragments, after checking for congruence. A total of 56 taxa were included in the analysis of the combined datasets, with one species of *Luisia* Gaudich., one species of *Saccolabium* Blume, two species of *Holcoglossum* Schltr. and two species of *Pomatocalpa* Breda used as the outgroups based on Liu et al. (2019) and Zhang et al. (2024). The nucleotide substitution model for the data matrix was estimated using jModeltest v2.1.6 (Posada 2008) and the evolutionary best fit model (GTR+F+I+G4) was selected using the corrected Akaike Information Criterion (AICc). Maximum likelihood (ML) and Bayesian inference (BI) methods were employed for phylogenetic tree reconstruction. The ML analysis was performed using IQ-TREE v1.4.2 (Nguyen et al. 2014) with branch support estimated using 2,000 replicates. The BI analysis was conducted using MrBayes v3.2.7a (Ronquist and Huelsenbeck 2003) with two separate Markov chain Monte Carlo (MCMC) chains (20,000,000 generations and sampled every 1,000 generations). The first 25% of the trees were discarded as burn-in, and the remaining trees were used to generate a majority-rule consensus tree. The resulted phylogenetic trees were visualized using Chiplot (Xie et al. 2023).

## ﻿Results

### ﻿Phylogenetic reconstruction

**Figure 1.**
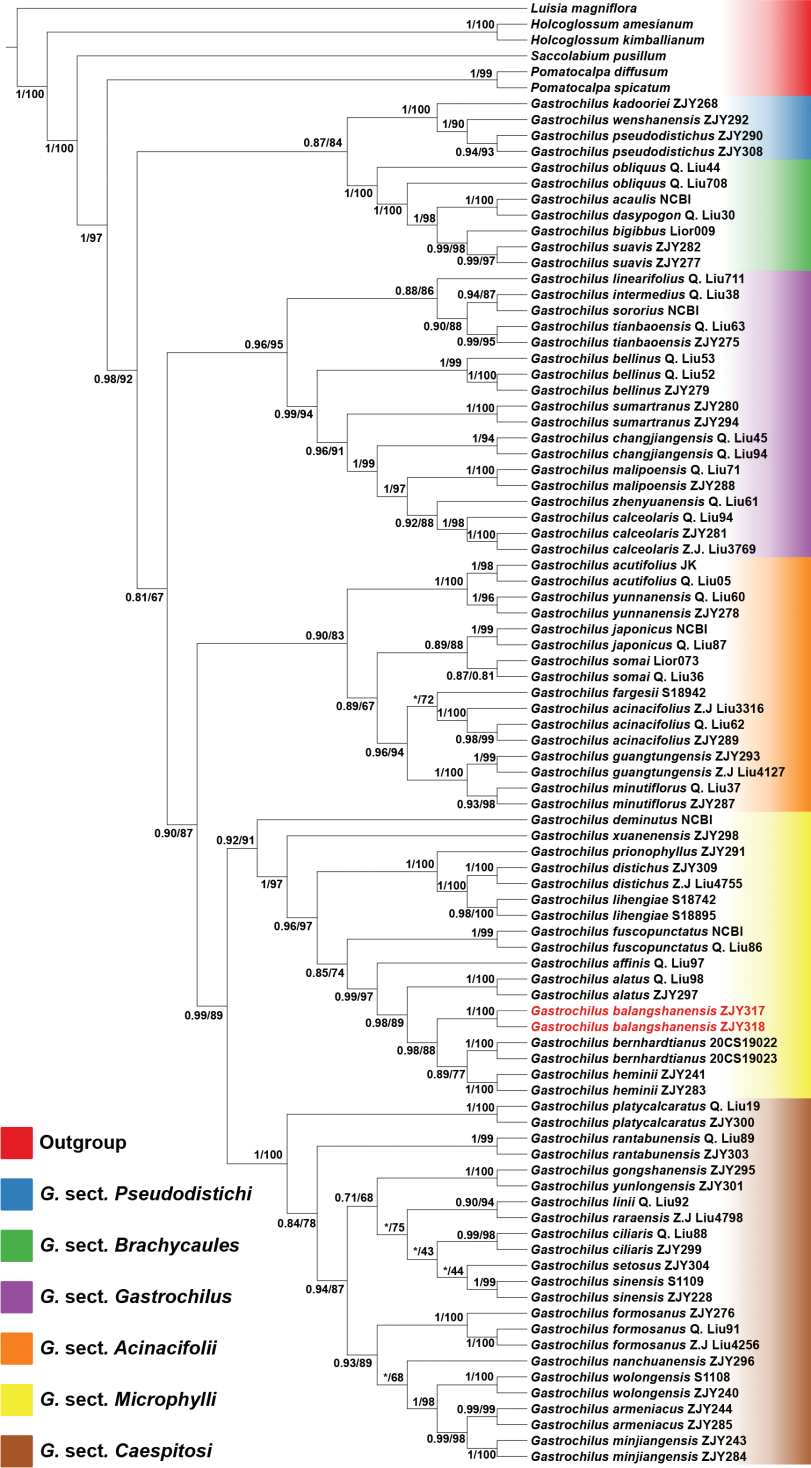
Maximum Likelihood phylogenetic tree of *Gastrochilus*, including 50 taxa, based on the combined nrITS and four-plastid (*mat*K, *psb*A–*trn*H, *psb*M–*trn*D, and *trn*L–F) marker dataset. Values before slash indicate Bayesian posterior probabilities and numbers after slash indicate ML bootstrap supports for major lineages. An asterisk (*) indicates that a node is not supported in the analysis. The two accessions of the inferred new species are highlighted in red, and colors of terminal nodes correspond to the six sections of *Gastrochilus* defined in Zhang et al. (2024).

**Figure 2.**
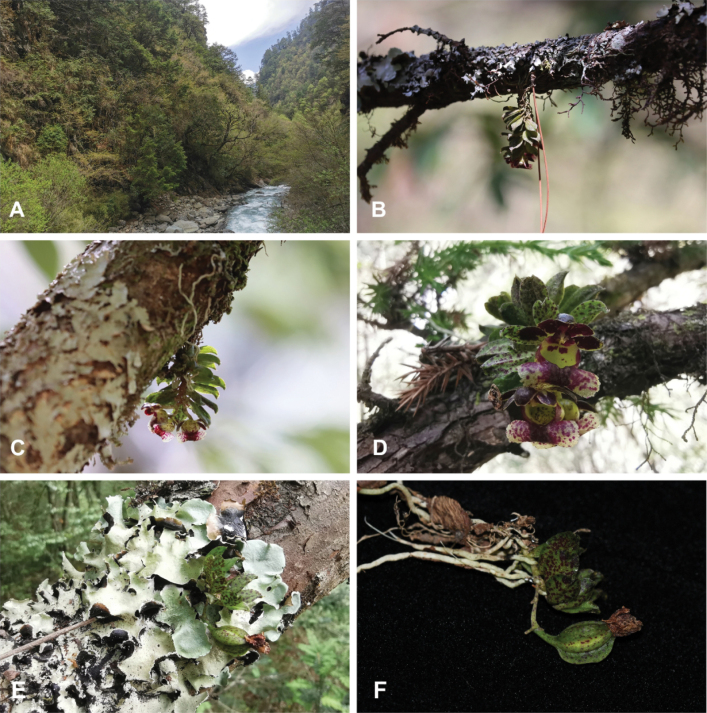
Habitat and habit of *Gastrochilusbalangshanensis* in situ **A** habitat **B–D** flowering plants of *G.balangshanensis* growing on tree trunks or branches **E, F** fruiting plants of *G.balangshanensis*. Photograph credits: **A–E** Yue-Hong Cheng **F** Jun-Yi Zhang.

The aligned nrITS matrix is 687 nucleotides long with 188 variable sites, and the combined four plastid markers matrix included 3,458 nucleotides in length with 185 variable sites, consists of 805 bp for *mat*K, 677 bp for *psb*A–*trn*H, 945 bp for *psb*M–*trn*D, and 1031 bp for *trn*L–F, respectively. The attributes of the five plastid markers are summarized in Appendix 1: Table A1. Both ML and BI analyses of the combined nrITS and four plastid markers matrix produced similar topologies (Fig. 1). The 50 taxa of *Gastrochilus* form a well-supported monophyletic group (BI/ML = 1/97, Fig. 1), which was subdivided into six well-supported section-specific clades (*G.* sects. *Gastrochilus*, *Pseudodistichi*, *Brachycaules*, *Acinacifolii*, *Microphylli*). The new species is resolved as distinct within section G.sect.Microphylli. The two accessions of *G.balangshanensis* were resolved as sisters to each other (BI/ML = 1/100, Fig. 1), clustering successively with *G.bernhardtianus* J.D.Ya & D.Z.Li and *G.heminii* M.Liao, B.Xu & Yue H.Cheng (Fig. 2; BI/ML = 0.98/88%).

Morphological comparison among *G.balangshanensis*, *G.heminii*, *G.bernhardtianus* and *G.affinis* is summarized in Table 1, further supporting the recognition of *Gastrochilusbalangshanensis* sp. nov.

**Table 1.** Morphological comparison of *Gastrochilusbalangshanensis* with three related species of G.sect.Microphylli.

Character	*G.balangshanensis*	*G.heminii*	*G.affinis*	*G.bernhardtianus*
Stem length	1.5–3.5 cm	3.0–6.5 cm	3.0–12.0 cm	ca. 5.0 cm
Leaf shape	nearly elliptic	narrowly oblong or oblong-falcate	oblong-lanceolate to subspathulate	oblong-lanceolate
No. of flowers per inflorescence	1 or 2	1 or 2	1–4	1 or 2
Peduncle length	0.8–1.2 cm	0.4–0.7 cm	1.5–2.0 cm	ca. 0.3 cm
Dorsal sepal	elliptic, ca. 5.6–6.4 × 4.8–5.2 mm, apex obtuse	elliptic-oblong, ca. 2.4 × 1.5 mm, apex obtuse	elliptic-oblong, 3.0–5 .0 × 1.0–1.3 mm, apex obtuse	elliptic, ca. 5.2 × 3.4 mm, apex obtuse
Lateral sepals	oblong, 5.0–5.8 × 4.0–4.4 mm, apex obtuse	narrowly oblong, 2.6 × 1.3 mm, apex obtuse	elliptic-ovate, 3.5–4.0 × 0.7–1.3 mm, apex obtuse	narrowly ovate, 5.5 × 2.8 mm, apex obtuse
Petals	oblong, 5.0–5.8 × 4.0–4.4 mm, apex obtuse	narrowly oblong, ca. 2.6 × 1.3 mm, apex obtuse	ovate-elliptic to elliptic, 3.0–4.0 × 1.0–1.3 mm, apex obtuse	narrowly oblong, ca. 5.2 × 2.7 mm, apex obtuse
Epichile (lip lamina)	reniform, 10.0–12.0 × 5.5–6.5 mm, central thickened purple-red mat with two inconspicuous ridges	reniform, 4.2–6.5 × 2.0–3.0 mm, central thickened purple-red mat with irregular folds	subtriangular, ca. 8.0 × 4.5 mm, central thickened purple-red mat with 2 median ridges from base to apex	transversely oblong, ca. 8.0 × 2.8 mm, central thickened yellow-green mat with 2 conic calli near its base
Hypochile (lip spur)	sub-hemispherical, 6.0–8.0 × 5.8–7.5 mm, dorsally compressed, obtuse-rounded at the apex	subconical or helmet-shaped, ca. 2.0–2.4 × 1.6–2.0 mm, dorsally compressed, splits into two conical sacs at the apex	obconical, 3.0–4.0 × 2.0–3.0 mm, dorsally compressed, shortly bifid at the apex	subconical, ca. 5.1 × 3.8 mm, dorsally compressed splits into two conical sacs at the apex

### ﻿Taxonomic treatment

#### ﻿ Gastrochilus balangshanensis

Taxon classificationPlantaeAsparagalesOrchidaceae

Jun Y.Zhang, B.Xu & Yue H.Cheng sp. nov.

13F08865-5F2F-5BAA-8F67-8B0AA45CAD56

urn:lsid:ipni.org:names:77349954-1

Figs 2A, B , 3

##### Type.

China • Sichuan: Wenchuan, Balangshan, Yinchangou, mixed coniferous forest, on tree branches, elev. ca. 2,260 m, in flower, 19 April 2023, *Jun-Yi Zhang & Yue-Hong Cheng ZJY185* (holotype: CDBI!; isotype: KUN!).

##### Diagnosis.

*Gastrochilusbalangshanensis* is most similar to *G.affinis*, but can be distinguished by its shorter stem (1.5–3.5 vs. 3.0–12.0 cm), nearly elliptic leaves (vs. oblong-lanceolate to subspathulate), larger sepals (5.6–6.4 × 4.8–5.2 vs. 3.0–5.0 × 1.0–1.3 mm) and petals (5.0–5.8 × 4.0–4.4 vs. 3.0–4.0 × 1.0–1.3 mm), reniform epichile (vs. subtriangular) and sub-hemispherical hypochile, obtuse-rounded at the apex (vs. hypochile obconical, subacute to obtuse and shortly bifid at apex).

**Figure 3.**
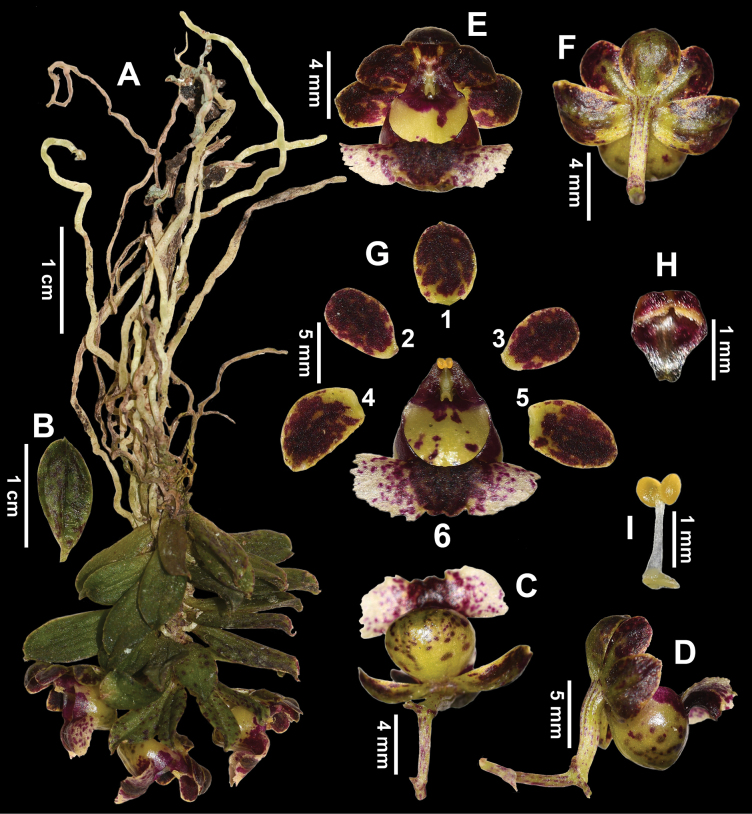
*Gastrochilusbalangshanensis***A** habit **B** leaf, abaxial view **C** raceme, front view **D** raceme, lateral view **E** flowers, front view **F** flowers, abaxial view **G1** dorsal sepal **G2, 3** petals **G4, 5** lateral sepals **G6** labellum **H** anther cap, ventral view **I** pollinarium with pollinia.

##### Description.

Epiphytic herb, monopodial, pendent, 1.5–3.5 cm tall. Roots vermiform, 4.0–6.0 cm long, ca. 1.6 mm in diameter. Stem unbranched, 0.5–2.5 cm long, ca. 1.5 mm in diameter. Leaves closely alternate, nearly elliptic, 0.9–1.5 × 0.4–0.8 cm, apex acute and with 1–2 lobules, lobes setaceous, with purplish-red spots. Inflorescence a raceme with 1 or 2 flowers; peduncle curved upward and thickened, 5.0–8.0 mm long, proximally covered with one sheath; floral bracts ovate-lanceolate, 0.8–1.2 cm long, apex acute. Flowers spreading, ca. 1.0 × 1.4 cm, pedicel and ovary connate, 6.0–9.0 mm long, sepals and petals heterochromatic on both surfaces, yellow-green with purplish-red spots on the outer side, purplish-red with yellow-green margin on the inner side. Dorsal and lateral sepals similar and equal in size, elliptic, 5.6–6.4 × 4.8–5.2 mm, apex obtuse. Petals oblong, 5.0–5.8 × 4.0–4.4 mm, apex acute, base narrowed. Labellum with a reniform epichile, revolute, white with purplish-red spots, 10.0–12.0 × 5.5–6.5 mm, margin erose, smooth and glabrous above, central thickened purple-red mat with two inconspicuous ridges; hypochile sub-hemispherical, yellowish-green with purplish-red spots, 6.0–8.0 × 5.8–7.5 mm, dorsally compressed, obtuse-rounded at the apex. Column cylindrical, ca. 1.5 mm long; viscidium yellow, ca. 0.7 × 0.4 mm; stipe white, ca. 1.1 mm long; anther cap purplish-red, ca. 1.5 × 1.3 mm, rostellum bilobed, lobes acuminate at the apex; pollinia 2, ca. 0.5 × 0.4 mm, yellow, subglobular, porate; stigma deeply sunken, inverted V-shaped, ca. 0.7 mm long, yellow. Capsule ellipsoid, 12.0–15.0 × 8.0–10.0 mm, green with sparse purplish-red spots, prominently 6-ribbed.

##### Distribution and habitat.

**Figure 4.**
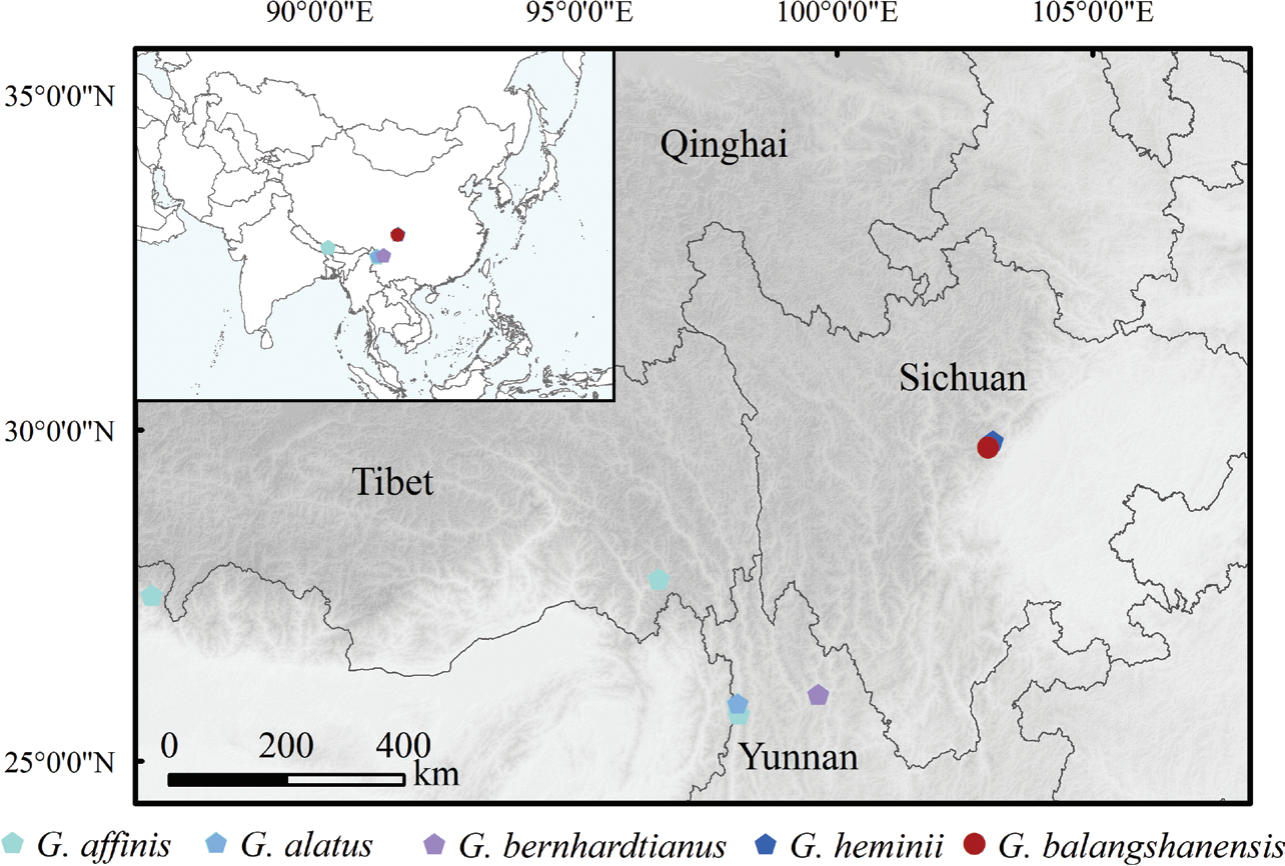
Distribution map of *Gastrochilusbalangshanensis* and four related species of G.sect.Microphylli.

*Gastrochilusbalangshanensis* is currently known only from Yinchanggou, Balang Mountain, Wenchuan County, Sichuan Province, Southwest China, part of the Hengduan Mountains (Fig. 4). It is epiphytic on tree trunks or branches in subalpine mixed coniferous forest dominated by Abiesfargesiivar.faxoniana (Rehder & E.H.Wilson) T.S.Liu, at an elevational range between 2200–2300 m, co-occurring with two terrestrial orchids, *Goodyerarecurva* Lindl. and *Calanthearcuata* Rolfe.

##### Phenology.

Flowering from March to April, during the dry season, fruiting from May to September.

##### Etymology.

Its specific epithet refers to the Balang Mountain, type locality of this new orchid. A Chinese name, “ba lang shan peng ju lan” (巴朗山盆距兰), is suggested here.

##### Additional specimens examined.

China • Sichuan: Wenchuan, Balangshan, Yinchangou, mixed coniferous forest, on tree trunk, elev. ca. 2300 m, in flower, 19 April 2023, *Jun-Yi Zhang & Yue-Hong Cheng ZJY186* (CDBI!); *ibid. loc.*, mixed coniferous forest, on tree branch, elev. ca. 2315 m, in fruit, 22 July 2024, *Jun-Yi Zhang & Yue-Hong Cheng ZJY204* (CDBI!).

##### Examined specimens of *Gastrochilusaffinis*.

India • Arunachal Pradesh: Lachong Valley, elev. ca. 2438 m, in flower, July 1897, *R. Pantling 444* (lectotype K). China • Xizang: Chayu, under forest, elev. ca. 2685 m, in flower, 24 August 2009, *Southeast Tibet Expedition Team SET-ET 717* (PE); China • Yunnan: Fugong, Jiakedi, east slope of Gaoligongshan, epiphytic on trunk, elev. ca. 2555 m, in flower, 16 May 2005, *X. H. Jin6984* (PE).

##### Examined specimens of *Gastrochilusbernhardtianus*.

China • Yunnan: Lijiang Prefecture, Yulong County, Yunshanping, elev. ca. 3308 m, in cold-temperate, evergreen conifer forest, in flower, 20 May 2020, *J.-D. Ya et al. 20CS19022* (holotype KUN).

##### Examined specimens of *Gastrochilusheminii*.

China • Sichuan: Wenchuan, Wolong, mixed coniferous forest, on tree trunk, elev. ca. 2640 m, in flower and fruit, 15 March 2022, *Min Liao & Yue-Hong Cheng ZJY143* (holotype CDBI!); Wenchuan County, Wolong, mixed coniferous forest, on tree trunk, elev. ca. 2640 m, in flower, 14 April 2022, *Min Liao, Jun-Yi Zhang & Yue-Hong Cheng ZJY167* (CDBI!).

## ﻿Discussion

The Hengduan Mountains (HDM) is part of the Tibeto-Himalayan region (THR), which possesses an ﻿exceptionally diverse flora (~12,800 seed plant species are recognized, Sun et al. 2017). We have previously discovered some new species of *Gastrochilus* in Wenchuan County, such as *G.wolongensis* Jun Y.Zhang, B.Xu & Yue H.Cheng (Zhang et al. 2022), *G.heminii* M.Liao, B.Xu & Yue H.Cheng (Liao et al. 2022), *G.armeniacus* Jun Y.Zhang, B.Xu & Yue H.Cheng (Zhang et al. 2024) and *G.minjiangensis* Jun Y.Zhang, B.Xu & Yue H.Cheng (Zhang et al. 2024). The discovery of *Gastrochilusbalangshanensis* reinforces the need for more in-depth botanical exploration in this region.

Based on a series of morphological characteristics, including leaf shape, leaf size and color markings, *Gastrochilusbalangshanensis* is most similar to *G.bernhardtianus* and *G.heminii*. However, based on overall floral characters, namely color, *G.balangshanensis* is most similar to *G.affinis*. This is possibly explained by parallel evolution.

Our phylogenetic analysis retrieved *Gastrochilusbalangshanensis* in a clade with *G.bernhardtianus* and *G.heminii*, both also endemic to the HDM. This supports the recognition of an overlooked center of endemism, possibly associated with rapid allopatric speciation in close mountain ranges within G.sect.Microphylli, which now numbers 11 species.

## Supplementary Material

XML Treatment for Gastrochilus balangshanensis

## ﻿Additional information

### Author ORCIDs

Jun-Yi Zhang https://orcid.org/0000-0001-8325-5954

Yue-Hong Cheng https://orcid.org/0000-0001-6263-0989

Min Liao https://orcid.org/0000-0001-6777-8579

Sen-Long Jin https://orcid.org/0000-0003-0200-4710

Hong-Qiang Lin https://orcid.org/0000-0001-8152-4911

Pan-Yan Yang https://orcid.org/0009-0004-0722-8953

Hai He https://orcid.org/0000-0001-5362-9177

Bo Xu https://orcid.org/0000-0003-3507-9321

### Data availability

All of the data that support the findings of this study are available in the main text.

## ﻿Appendix 1

**Table A1.** Information on the DNA markers used in phylogenetic reconstruction of *Gastrochilus*.

Marker	Length (bp)	Variable sites (bp)	Primer Sequence (5’to3’)	Origin
nrITS	687	188	GGAAGGAGAAGTCGTAACAAGG	Thiv et al. 1999
			CTTTTCCTCCGCTTATTGATATG	Thiv et al. 1999
*psb*A–*trn*H	677	37	GTTATGCATGAACGTAATGCTC	Sang et al. 1997
			CGCGCATGGTGGATTCACAAATC	Sang et al. 1997
*mat*K	805	28	CGATCTATTCATTCAATATTTC	Sun et al. 1994
			TCTAGCACACGAAAGTCGA	Sun et al. 1994
*psb*M–*trn*D	945	65	GCGGTAGGAACTAGAATAAATAG	Zuo et al. 2011
			GGGATTGTAGTTCAATTGGT	Zuo et al. 2011
*trn*L-F	1031	55	CGAAATCGGTAGACGCTACG	Taberlet et al. 1991
			ATITGAACTGGTGACACGAG	Taberlet et al. 1991

**Table A2.** Voucher information and GenBank accession numbers of DNA sequences of *Gastrochilus* used in phylogenetic inference.

Taxa	Voucher	Location	nrITS	*mat*K	*psb*A–*trn*H	*trn*L–F	*psb*M–*trn*D
*G.acaulis* (Lindl.) Kuntze	—	—	KM583455	KM583465	—	—	—
*G.acinacifolius* Z.H.Tsi	*Z. J. Liu 3316*	CHINA	KJ733412	KJ733569	KJ733492	KJ733649	—
*G.acinacifolius* Z.H.Tsi	*Q. Liu 62*	CHINA: Hainan (Baomeiling)	MK357118	MK357138	MK357160	MK357208	MK357216
*G.acinacifolius* Z.H.Tsi	*ZJY289*	CHINA: Yunnan (Malipo)	OQ566796	OQ575562	OQ575590	OQ575530	OQ575619
*G.acutifolius* (Lindl.) Kuntze	*Q. Liu 05*	CHINA: Yunnan (Jingmai)	—	MK357140	MK357162	—	MK357230
*G.acutifolius* (Lindl.) Kuntze	JK-DEBCR-mat-31(JK)	—	MW475270	MW433889	—	—	—
*G.affinis* (King & Pantl.) Schltr.	*Q. Liu 97*	CHINA: Yunnan (Fugong)	—	MK357141	MK357163	—	MK357141
*G.alatus* X.H.Jin & S.C.Chen	*Q. Liu 98*	CHINA: Yunnan (Bingzhongluo)	—	—	—	—	MK357228
*G.alatus* X.H.Jin & S.C.Chen	*ZJY297*	CHINA: Yunnan	—	OQ575563	OQ575591	OQ575531	OQ575620
*G.armeniacus* Jun Y.Zhang, B.Xu & Yue H.Cheng	*ZJY244*	CHINA: Sichuan (Wenchuan)	OP348889	OP373113	OP373119	OP373128	OP373123
*G.armeniacus* Jun Y.Zhang, B.Xu & Yue H.Cheng	*ZJY285*	CHINA: Sichuan (Wenchuan)	OP348888	OP373114	OP373118	OP373127	OP373124
*G.balangshanensis* Jun Y.Zhang, B.Xu & Yue H.Cheng	*ZJY317*	CHINA: Sichuan (Wenchuan)	PP960151	PP944493	PP944495	PP944499	PP944497
*G.balangshanensis* Jun Y.Zhang, B.Xu & Yue H.Cheng	*ZJY318*	CHINA: Sichuan (Wenchuan)	PP960152	PP944494	PP944496	PP944500	PP944498
*G.bellinus* (Rolfe. F.) Kuntze	*Q. Liu 52*	CHINA: Yunnan (Lancang)	KY966597	KY966884	—	—	—
*G.bellinus* (Rolfe. F.) Kuntze	*Q. Liu 53*	CHINA: Yunnan (Mengsong)	MK357123	MK357142	MK357164	MK357202	MK357241
*G.bellinus* (Rolfe. F.) Kuntze	*ZJY279*	CHINA: Yunnan (Mengla)	OQ566799	OQ575564	OQ575592	OQ575534	OQ575621
*G.bernhardtianus* J.D.Ya & D.Z.Li	*20CS19022*	CHINA: Yunnan (Lijiang)	OR073404	20CS19022	20CS19022	20CS19022	20CS19022
*G.bernhardtianus* J.D.Ya & D.Z.Li	*20CS19023*	CHINA: Yunnan (Lijiang)	OR073405	20CS19023	20CS19023	20CS19023	20CS19023
*G.bigibbus* (Rchb. f. ex Hook. f.) Kuntze	Li 009	—	—	MN124439	MN124439	MN124439	MN124439
*G.calceolaris* D.Don	*Z. J. Liu 3769*	CHINA	KF545874	KF545885	KF545865	—	KF545896
*G.calceolaris* D.Don	*Q. Liu 94*	CHINA: Yunnan (Jingdong)	MK357126	MK357144	MK357169	MK357205	MK357233
*G.calceolaris* D.Don	*ZJY281*	CHINA: Yunnan (Malipo)	—	OQ575565	OQ575593	OQ575535	OQ575622
*G.changjiangensis* Q.Liu & M.Z.Huang	*Q. Liu 45*	CHINA: Hainan (Changjiang)	MK357124	—	MK357166	—	MK357236
*G.ciliaris* F.Maekawa	*Q. Liu 88*	CHINA: Nursery of Management Office of Yushan Scenic Area, Fuzhou	—	MK357148	MK357173	—	MK357225
*G.ciliaris* F.Maekawa	*ZJY299*	CHINA: Taiwan	—	OQ575566	OQ575594	—	OQ575623
*G.dasypogon* (Lindl.) Kuntze	*Q. Liu 30*	CHINA: Hainan (Baomeiling)	MK357129	MK357149	MK357181	MK357197	MK357219
*G.deminutus* J.M.H.Shaw	—	—	KY966600	KY966887	—	—	—
*G.distichus* (Lindl.) Kuntze	*Z. J. Liu 4755*	CHINA	KJ733414	KJ733571	KJ733494	KJ733651	—
*G.distichus* (Lindl.) Kuntze	*ZJY309*	CHINA: Yunnan (Malipo)	OQ566800	OQ575567	OQ575595	OQ575536	—
*G.fargesii* (Kraenzl.) Schltr.	*20HT3548*	CHINA: Chongqing	OR073405	S18942	S18942	S18942	S18942
*G.formosanus* (Hayata) Hayata	*Z. J. Liu 4256*	CHINA	KJ733416	KJ733573	KJ733495	KJ733653	—
*G.formosanus* (Hayata) Hayata	*Q. Liu 91*	CHINA: Yunnan (Gongshan)	—	—	MK357174	—	MK357226
*G.formosanus* (Hayata) Hayata	*ZJY276*	CHINA: Hubei (Enshi)	OQ566801	OQ575568	OQ575596	OQ575537	OQ575624
*G.fuscopunctatus* (Hayata) Hayata	*Q. Liu 86*	CHINA: Taiwan (Hualian)	—	MK357150	MK357171	MK357192	MK357231
*G.fuscopunctatus* (Hayata) Hayata*	—	—	—	KX871233	KX871233	KX871233	KX871233
*G.gongshanensis* Z.H.Tsi	*ZJY295*	CHINA: Yunnan (Malipo)	—	OQ575569	OQ575597	OQ575538	OQ575625
*G.guangtungensis* Z.H.Tsi	*Z. J. Liu 4127*	CHINA	KJ733417	KJ733574	KJ733496	KJ733654	—
*G.guangtungensis* Z.H.Tsi	*ZJY293*	CHINA: Yunnan	OQ566802	OQ575570	OQ575598	OQ575539	OQ575626
*G.heminii* M.Liao, B.Xu & Yue.H.Cheng	*ZJY241*	CHINA: Sichuan (Wenchuan)	ON286752	ON331126	ON331128	ON331130	ON331132
*G.heminii* M.Liao, B.Xu & Yue.H.Cheng	*ZJY283*	CHINA: Sichuan (Wenchuan)	ON286753	ON331127	ON331129	ON331131	ON331133
*G.intermedius* (Griff. ex Lindl.) Kuntze	*Q. Liu 38*	CHINA: Yunnan (Yinchang)	MK357121	MK357151	MK357172	MK357190	MK357213
*G.japonicus* (Makino) Schltr.	*Q. Liu 87*	CHINA: Taiwan (Pingdong)	KF545875	KF545886	KF545866	KF545897	—
*G.japonicus* (Makino) Schltr.	—	—	—	KX871236	KX871236	KX871236	KX871236
*G.kadooriei* Kumar et al.	*ZJY268*	CHINA: Yunnan (Malipo)	OQ566803	OQ575571	OQ575599	OQ575540	OQ575627
*G.lihengiae* J.D.Ya, H.Jiang & D.Z.Li	*22CS21828*	CHINA: Yunnan (Gongshan)	OR073408	S18742	S18742	S18742	S18742
*G.lihengiae* J.D.Ya, H.Jiang & D.Z.Li	*23CS24145*	CHINA: Yunnan (Gongshan)	OR073408	S18895	S18895	S18895	S18895
*G.linearifolius* Z.H.Tsi & Garay	*Q. Liu 711*	MYANMA: Hkakaborazi National Park	MK357133	MK357136	MK357187	MK357194	MK357229
*G.linii* Ormerod	*Q. Liu 92*	CHINA: Nursery of Management Office of Yushan Scenic Area, Fuzhou	—	MK357152	MK357176	MK357198	MK357224
*G.malipoensis* X.H.Jin & S.C.Chen	*Q. Liu 71*	CHINA: Yunnan (Nanwenghe)	—	MK357147	MK357177	MK357200	MK357235
*G.malipoensis* X.H.Jin & S.C.Chen	*ZJY288*	CHINA: Yunnan (Malipo)	OQ566804	OQ575572	OQ575600	OQ575541	OQ575628
*G.matsuran* (Makino) Schltr.	—	—	KT338700	—	—	—	—
*G.minjiangensis* Jun Y.Zhang, B.Xu & Yue H.Cheng	*ZJY243*	CHINA: Sichuan (Wenchuan)	OP348887	OP373112	OP373117	OP373126	OP373122
*G.minjiangensis* Jun Y.Zhang, B.Xu & Yue H.Cheng	*ZJY284*	CHINA: Sichuan (Wenchuan)	OQ566806	—	—	OQ575542	—
*G.minutiflorus* Aver.	*Q. Liu 37*	CHINA: Yunnan (Malipo)	—	MK357153	MK357179	—	MK357215
*G.minutiflorus* Aver.	*ZJY287*	CHINA: Yunnan (Malipo)	OQ566807	OQ575573	OQ575601	OQ575544	OQ575629
*G.nanchuanensis* Z.H.Tsi	*ZJY296*	CHINA: Chongqing (Nanchuan)	OQ566808	OQ575574	OQ575602	OQ575545	OQ575630
G.obliquusvar.obliquus (Lindl.) Kuntze	*Q. Liu 708*	MYANMA: Hponkanrazi Wildlife Sanctuary	MK357131	MK357137	KJ733498	KJ733656	MK357211
G.obliquusvar.obliquus (Lindl.) Kuntze	*Q. Liu 44*	CHINA: Yunnan (Yinchang)	MK357130	MK357154	MK357182	MK357195	MK357218
*G.suavis* Seidenf.	*ZJY277*	CHINA: Yunnan	OQ566809	OQ575575	OQ575603	OQ575546	OQ575631
*G.suavis* Seidenf.	*ZJY282*	CHINA: Yunnan (Maguan)	OQ566810	OQ575576	OQ575604	OQ575547	OQ575632
*G.platycalcaratus* (Rolfe) Schltr.	*Q. Liu 19*	CHINA: Yunnan (Yinchang)	MK357122	—	MK357175	—	MK357222
*G.platycalcaratus* (Rolfe) Schltr.	*ZJY300*	CHINA: Yunnan (Mengla)	OQ566811	—	OQ575605	OQ575548	OQ575633
*G.prionophyllus* H.Jiang, D.P.Ye & Q.Liu	*ZJY291*	CHINA: Yunnan	OQ566812	OQ575577	OQ575606	OQ575549	OQ575634
*G.pseudodistichus* (King & Pantl.) Schltr.	*ZJY308*	CHINA: Yunnan (Malipo)	OQ566813	OQ575578	OQ575607	OQ575550	OQ575635
*G.pseudodistichus* (King & Pantl.) Schltr.	*ZJY290*	CHINA: Yunnan (Maguan)	OQ566814	OQ575579	OQ575608	OQ575551	OQ575636
*G.rantabunensis* S.Chow ex T.P.Lin	*Q. Liu 89*	CHINA: Taiwan (Taipei)	—	MK357155	MK357184	MK357193	MK357223
*G.rantabunensis* S.Chow ex T.P.Lin	*ZJY303*	CHINA: Taiwan	OQ566815	OQ575580	OQ575609	OQ575552	OQ575637
*G.raraensis* Fukuy.	*Z. J. Liu 4798*	CHINA	KJ733420	KJ733577	KJ733499	KJ733657	—
*G.setosus* Aver. & Vuong	*ZJY304*	Vietnam	OQ566816	OQ575581	OQ575610	OQ575553	—
*G.sinensis* Z.H.Tsi	*ZJY228*	CHINA: Zhejiang (Hangzhou)	OM985813	OK042953	OK172399	OK172401	OQ575646
*G.sinensis* Z.H.Tsi	*S1109*	CHINA: Sichuan (Wenchuan)	OP348890	OP373115	OP373120	OP373129	OP373125
*G.somai* (Hayata) Hayata	*Q. Liu 36*	CHINA: Fujian (Pingnan)	MK357128	—	MK357180	—	MK357220
*G.somai* (Hayata) Hayata	Li 073	—	—	MN124436	MN124436	MN124436	MN124436
*G.sororius* Schltr.	—	—	ERR7622420	ERR7622420	ERR7622420	ERR7622420	
*G.wenshanensis* Q.Liu, D.P.Ye & X.H.Jin	*ZJY292*	CHINA: Yunnan (Maguan)	OQ566820	OQ575586	OQ575615	OQ575558	OQ575642
*G.sumartranus* J.J.Sm.	*ZJY280*	Vietnam	OQ566818	OQ575583	OQ575612	OQ575555	OQ575639
*G.sumartranus* J.J.Sm.	*ZJY294*	Vietnam	OQ566819	OQ575584	OQ575613	OQ575556	OQ575640
*G.tianbaoensis* Q.Liu & Y.H.Tan	*ZJY275*	CHINA: Yunnan (Malipo)	—	OQ575585	OQ575614	OQ575557	OQ575641
*G.tianbaoensis* Q.Liu & Y.H.Tan	*Q. Liu 63*	CHINA: Yunnan (Malipo)	MK357120	MK357157	MK357186	MK357207	MK357214
*G.wolongensis* Jun Y.Zhang, B.Xu & Yue H.Cheng	*ZJY240*	CHINA: Sichuan (Wenchuan)	OM985810	OK172400	OK172402	OK172404	OK172403
*G.wolongensis* Jun Y.Zhang, B.Xu & Yue H.Cheng	*S1108*	CHINA: Sichuan (Wenchuan)	OM985811	OM974209	OM974211	OM974210	OQ575647
*G.xuanenensis* Z.H.Tsi	*ZJY298*	CHINA: Guizhou	—	OQ575587	OQ575616	OQ575559	OQ575643
*G.yunlongensis* W.H.Rao, L.J.Chen & Z.J.Liu	*ZJY301*	CHINA: Yunnan	—	OQ575588	OQ575617	OQ575560	OQ575644
*G.yunnanensis* Schltr.	*Q. Liu 60*	CHINA: Yunnan (Mengsong)	MK165469	MK357158	MK357185	—	MK357212
*G.yunnanensis* Schltr.	*ZJY278*	CHINA: Yunnan	OQ566821	OQ575589	OQ575618	OQ575561	OQ575645
*G.zhenyuanensis* Q.Liu & D.P.Ye	*Q. Liu 61*	CHINA: Yunnan (Zhenyuan)	MK357127	MK357146	MK357168	MK357199	MK357237
*Holcoglossumamesianum* (Rchb. F.) Christenson	*X.H. Jin 004/006*	CHINA	HQ404389	JF763779	HQ404439	—	—
*H.kimballianum* (Rchb. F.) Garay	*X.H. Jin 017*	CHINA	HQ452901	JF763787	HQ404452	—	—
*Luisiamagniflora* Z.H.Tsi et S.C.Chen	*Z. J. Liu 3444*	CHINA	KJ733426	KJ733583	KJ733505	—	—
*Pomatocalpadiffusum* Breda	*TBG145837*	INDONESIA	AB217576	AB217752	—	EF670432	—
*P.spicatum* Breda	*Z. J. Liu 4589*	CHINA	KJ733438	KJ733595	KJ733518	KJ733675	—
*Saccolabiumpusillum* Blume	*TBG144220*	INDIA	AB217580	AB217756	—	—	—

## References

[B1] Beentje H (2016) The Kew plant glossary: an illustrated dictionary of plant terms, 2^nd^ edn. Kew Publishing, Kew, 184 pp.

[B2] Chen WS Lei M Ma CB Jin XH Wang XL (2022) *Gastrochilusxizangensis* (Aeridinae, Vandeae, Orchidaceae), a new species from Xizang, China. Phytotaxa 566(2): 219–226. 10.11646/phytotaxa.566.2.6

[B3] Dey S Phom L Bhattacharjee A Moaakum Eshuo K (2022) *Gastrochiluspseudocalceolaris*, a new species of epiphytic orchid from India. Phytotaxa 574(4): 295–300. 10.11646/phytotaxa.574.4.5

[B4] Don D (1825) Prodromus Florae Nepalensis. J. Gale, London, 256 pp.

[B5] Govaerts R Campacci MA Baptista DH Baptista PJ George A Kreutz K Wood JJ (2021) World Checklist of Orchidaceae. The Board of Trustees of the Royal Botanic Gardens, Kew. http://www.kew.org/wcsp/monocots/ [Accessed 13 August 2021]

[B6] Katoh K Standley DM (2013) MAFFT multiple sequence alignment software version 7: Improvements in performance and usability. Molecular Biology and Evolution 30(4): 772–780. 10.1093/molbev/mst010 23329690 PMC3603318

[B7] Lee CT Wu JH Wang YQ Hsieh SI (2023) *Gastrochilusyehii* sp. nov. (Orchidaceae) from Taiwan. Phytotaxa 587: 053–058. 10.11646/phytotaxa.587.1.7

[B8] Liao M Cheng YH Zhang JY Feng Y Liu GY Ye P Jin SL Lin HQ Xu B (2022) *Gastrochilusheminii* (Orchidaceae, Epidendroideae), a new species from Sichuan, China, based on molecular and morphological data. PhytoKeys 215: 95–106. 10.3897/phytokeys.215.91061 36761093 PMC9836483

[B9] Liu Q Song Y Jin XH Gao JY (2019) Phylogenetic relationships of *Gastrochilus* (Orchidaceae) based on nuclear and plastid DNA data. Botanical Journal of the Linnean Society 189(3): 228–243. 10.1093/botlinnean/boy084

[B10] Liu Q Wu XF Zhou SS Li JW Jin XH (2023) New species and record of *Gastrochilus* (Orchidaceae, Aeridinae) from China and Laos. Phytotaxa 585(3): 210–224. 10.11646/phytotaxa.585.3.3

[B11] Nguyen LT Schmidt HA Von-Haeseler A Minh BQ (2014) IQ-TREE: A fast and effective stochastic algorithm for estimating Maximum-Likelihood phylogenies. Molecular Biology and Evolution 32(1): 268–274. 10.1093/molbev/msu300 25371430 PMC4271533

[B12] Nguyen VC Averyanov LV Maisak TV Nguyen TLT Nguyen VK Truong BV (2022) *Gastrochiluspankajkumarii*, (Aeridinae, Epidendroideae, Orchidaceae) a new lithophytic orchid from southern Vietnam. Taiwania 67: 35–39. 10.6165/tai.2022.67.35

[B13] Posada D (2008) jModelTest: Phylogenetic model averaging. Molecular Biology and Evolution 25(7): 1253–1256. 10.1093/molbev/msn083 18397919

[B14] Pridgeon AM Cribb PJ Chase MW Rasmussen FN (2014) Genera orchidacearum: Epidendroideae, volume 6, Part 3. Oxford University Press, Oxford, 544 pp.

[B15] Rao WH Liu ZJ Zhang GQ Chen XH Huang J Chen GZ Chen LJ (2019) A new epiphytic species of *Gastrochilus* (Orchidaceae: Epidendroideae) from Yunnan, China. Phytotaxa 413(4): 296–300. 10.11646/phytotaxa.413.4.5

[B16] Ronquist F Huelsenbeck JP (2003) MrBayes 3: Bayesian phylogenetic inference under mixed models. Bioinformatics 19(12): 1572–1574. 10.1093/bioinformatics/btg180 12912839

[B17] Sang T Crawford D Stuessy T (1997) Chloroplast DNA phylogeny, reticulate evolution, and biogeography of *Paeonia* (Paeoniaceae). American Journal of Botany 84(8): 1120–1120. 10.2307/2446155 21708667

[B18] Sun Y Skinner D Liang G Hulbert SH (1994) Phylogenetic analysis of *Sorghum* and related taxa using internal transcribed spacers of nuclear ribosomal DNA. Theoretical and Applied Genetics 89(1): 26–32. 10.1007/BF00226978 24177765

[B19] Sun H Zhang J Deng T Boufford DE (2017) Origins and evolution of plant diversity in the Hengduan Mountains, China. Plant Diversity 39(4): 161–166. 10.1016/j.pld.2017.09.004 30159507 PMC6112316

[B20] Taberlet P Gielly L Pautou G Bouvet J (1991) Universal primers for amplification of three non-coding regions of chloroplast DNA. Plant Molecular Biology 17(5): 1105–1109. 10.1007/BF00037152 1932684

[B21] Thiers B (2021) Index Herbariorum: a global directory of public herbaria and associated staff. New York Botanical Garden’s Virtual Herbarium. http://sweetgum.nybg.org/science/ih

[B22] Thiv M Stuwe L Kadereit JW (1999) The phylogenetic relationships and evolution of the Canarian laurel forest endemic *Ixanthusviscosus* (Aiton) Griseb. (Gentianaceae): Evidence from *mat*K and ITS sequences, and floral morphology and anatomy. Plant Systematics and Evolution 218(3–4): 299–317. 10.1007/BF01089233

[B23] Tsi ZH (1996) A preliminary revision of *Gastrochilus* (Orchidaceae). Guihaia 16: 123–154.

[B24] Tsi ZH (1999) *Gastrochilus* D. Don. In: Tsi ZH (Ed.) Flora Reipublicae Popularis Sinicae, Vol.19. Science Press, Beijing, 399–420.

[B25] Wu XF Ye DP Pan B Lin XQ Jiang H Liu Q (2019) Validation of *Gastrochilusprionophyllus* (Vandeae, Orchidaceae), a new species from Yunnan Province, China. PhytoKeys 130: 161–169. 10.3897/phytokeys.130.34555 31534404 PMC6728340

[B26] Xie JM Chen YR Cai GJ Cai RL Hu Z Wang H (2023) Tree visualization by one Table (tvBOT): A web application for visualizing, modifying and annotating phylogenetic trees. Nucleic Acids Research 51(W1): W587–W592. 10.1093/nar/gkad359 PMC10320113 37144476

[B27] Ya JD Wang WT Liu YL Jiang H Han ZD Zhang T Huang H Cai J Li DZ (2023) Five new and noteworthy species of Epidendroideae (Orchidaceae) from southwestern China based on morphological and phylogenetic evidence. PhytoKeys 235: 211–236. 10.3897/phytokeys.235.111230 38033625 PMC10682981

[B28] Zhang JY Cheng YH Liao M Jin SL Qu CM Tan YC Plenković-Moraj A Xu B (2022) *Gastrochiluswolongensis* (Orchidaceae): A new species from Sichuan, China, based on molecular and morphological data. Ecosystem Health and Sustainability 8(1): 2101546. 10.1080/20964129.2022.2101546

[B29] Zhang JY Cheng YH Liao M Feng Y Jin SL He TM He H Xu B (2024) A new infrageneric classification of *Gastrochilus* (Orchidaceae: Epidendroideae) based on molecular and morphological data. Plant Diversity 46(4): 435–447. 10.1016/j.pld.2023.08.001 39280969 PMC11390603

[B30] Zhou ZH Shi RH Zhang Y Jin XH (2021) Orchid diversity in China: Recent discoveries. Plant Diversity 43(5): 341–342. 10.1016/j.pld.2021.07.004 34816059 PMC8591207

[B31] Zhou CY Zeng MY Wu YW Li MH (2024) *Gastrochiluspseudodresslerii* (Orchidaceae; Vandeae), a new species from China: evidence from morphological and DNA analysis. Phytotaxa 634: 071–078. 10.11646/phytotaxa.634.1.6

[B32] Zuo YJ Chen ZJ Kondo K Funamoto T Wen J Zhou SL (2011) DNA barcoding of *Panax* species. Planta Medica 77(2): 182–187. 10.1055/s-0030-1250166 20803416

